# Ivermectin treatment failure on four Irish dairy farms

**DOI:** 10.1186/s13620-019-0142-8

**Published:** 2019-05-15

**Authors:** James O’Shaughnessy, Yvonne Drought, John Lynch, Marian Denny, Christine Hurley, William Byrne, Mícheál Casey, Theo de Waal, Maresa Sheehan

**Affiliations:** 1 Central Veterinary Research Laboratory, Department of Agriculture, Food and the Marine Laboratories, Backweston, Celbridge, Co. Kildare Ireland; 2 School of Veterinary Medicine, University College Dublin, Belfield, Dublin 4, Ireland; 3 Archersfield Veterinary Clinic, Archersfield, Castle Rd, Kilkenny, Ireland; 4 Department of Agriculture, Food and the Marine, Kilkenny Regional Veterinary Laboratory, Hebron Road, Kilkenny, Ireland

**Keywords:** Anthelmintics, Cattle, *Cooperia*, Nematodes, *Ostertagia*, Resistance

## Abstract

We report on the use of the faecal egg count reduction test to evaluate the performance of ivermectin in treating gastrointestinal nematode infections in first grazing season (FGS) calves on four dairy farms in Co. Kilkenny, Ireland. On each farm, FGS calves were injected subcutaneously with ivermectin in accordance with their live weight (day 0). Calves were individually faecal sampled on both day 0 and day 14. Faecal egg counts were determined using the Mini-FLOTAC technique. Composite faecal cultures for each farm were performed on each sampling occasion. The faecal egg count reductions (mode) ranged from 17.3–80.2% with the lower 95% confidence limit ranging from 3.1–72.3% on the four farms, respectively. Ivermectin-resistant nematodes were detected on all farms, with evidence of *Ostertagia* resistance on one farm. This study highlights the urgent need for Irish producers to reappraise their parasite control practices.

**Electronic supplementary material:**

The online version of this article (10.1186/s13620-019-0142-8) contains supplementary material, which is available to authorized users.

## Introduction

Given the increase in the number of reports of the detection of anthelmintic-resistant nematodes in cattle [1–3], there is a clear need to establish the efficacy of commonly used anthelmintics on Irish cattle farms so as to ensure that neither animal welfare nor performance is compromised.

Of the anthelmintics available to beef and dairy producers, macrocyclic lactones (Group 3 -MLs) such as ivermectin are a popular choice [4, 5] and their popularity with producers can be readily explained by both their ease of use (e.g. available as pour-ons, low dose volume) and their persistence of activity against gastrointestinal nematodes [6, 7]. However, concerns exist over their long term sustainability and resistance to them has already been reported on two Irish beef research farms [8].

Although the controlled efficacy test is regarded as the gold standard for determining anthelmintic efficacy [9], the faecal egg count reduction test (FECRT) is more widely employed as it does not involve the slaughter of potentially expensive animals. The guidelines for conducting the FECRT were devised chiefly for sheep [10] but they can also be used for other species such as cattle, horse and pigs. Using this test, anthelmintic resistance (AR) is classified as occurring when the FECR is less than 95% with the lower 95% confidence limit being less than 90%. Resistance is only suspected when just one of these conditions are met.

In this study, we report on the use of the FECRT to evaluate the performance of ivermectin in treating gastrointestinal nematode (GIN) infections in first grazing season (FGS) calves on four dairy farms in Co. Kilkenny, Ireland.

## Materials and methods

On each of four dairy farms, a minimum of 12 FGS Holstein Friesian female calves which had not received any prior anthelmintic treatment and were on pasture for a minimum of eight weeks, were randomly selected and then injected subcutaneously with ivermectin (Ivomec Classic Injection for Cattle and Sheep 10 mg/ml, Boehringer Ingelheim Vetmedica GmbH) by a private veterinary practitioner using an injection syringe. Calves were treated in accordance with their live weight (day 0) as determined with the aid of a weigh tape at a dosage rate of 1 ml of the product per 50 kg of live weight. All dosage volumes were rounded up to the nearest millilitre. Calves were individually rectal faecal sampled on both day 0 and day 14. Faecal egg counts were determined using the Mini-FLOTAC technique [11] (limit of detection of five eggs per gram of faeces (EPG)). Data were analysed using the ‘shiny-eggCounts’ package (http://shiny.math.uzh.ch/user/furrer/shinyas/shiny-eggCounts/). Composite faecal cultures for each farm were performed (2 g of faeces per calf) on each sampling occasion to determine the composition of nematode genera in each treatment group. Cultures were incubated at 27 °C for eight days and 100 L_3_ larvae per culture were identified to genus level using standard identification keys [12] on recovery. All L_3_ larvae were identified when counts were less than 100. A questionnaire (see Additional file 1) was also prepared so that details of parasite control practices and grazing management strategies for FSG calves on each farm could be recorded.

## Results and discussion

Across all farms, the mean (arithmetic) FEC on day 0 did not exceed 100 EPG (Table 1). The FEC reductions (mode) ranged from 17.3–80.2% with the lower 95% confidence limit ranging from 3.1–72.3% on the four farms, respectively. *Cooperia* L_3_ larvae were detected in all four post-treatment faecal cultures (Table 2), with both *Ostertagia* and *Bunostomum* L_3_ larvae also being identified in the post-treatment faecal culture from Farm 2.

**Table 1:** Details of the calves sampled and the FECRT values following ivermectin treatment

Calf details (day 0)					% Reduction in faecal egg count on day 14		
Farm	Sampling date	No. calves sampled	Mean (s.d.) calf live weight (kg)	Pre-treatment FEC (arithmetic mean)	95% CI	Median	Mode
1	June 28th 2016	15	134 (18.6)	97	3.1–29.3	16.4	17.3
2	June 28th 2016	14	163 (25.3)	90	72.3–84.9	79.4	80.2
3	July 25th 2016	14	138 (14.8)	46	4.2–41.1	23.4	25.9
4	July 27th 2016	12	138 (10.9)	37	54.5–80.9	70	71.7

*FECRT* Faecal egg count reduction test

*CI* Confidence interval

*S.D* Standard deviation

**Table 2:** Nematode genera identified in composite pre- and post-treatment faecal cultures on four farms

		Farm			
		1	2	3	4
Day	Nematode genus and no. of L_3_ larvae per faecal culture				
0	*Cooperia*	29	26	37	36
	*Ostertagia*	71	73	63	62
	*Trichostrongylus*	0	1	0	2
	*Bunostomum*	0	0	0	0
14	*Cooperia*	31	30	47	25
	*Ostertagia*	0	8	0	0
	*Trichostrongylus*	0	0	0	0
	*Bunostomum*	0	1	0	0

With regard to the survey conducted, none of the farmers had previously used either faecal egg counting or calf growth rates as a guide to determine the need for anthelmintic treatment. All four farmers reported using the ‘dose and move’ system, with all calves in the group being treated at the same time with anthelmintics prior to moving to silage aftermath. All anthelmintic treatments given to FGS calves in the previous year were for preventive purposes only using an avermectin-based product, with the first treatment on two of the farms given at six weeks post turnout. Three of the farmers reported that they treat FGS calves a minimum of three times in the first year, while three also use the same parcel of grazing land each year for FGS calves. On two farms, calves were turned out to pasture in May while on the other two farms the month of turnout was March and April, respectively.

Using standardised criteria for defining the occurrence of AR [10], we report the presence of ivermectin resistant nematodes on all four study farms. None of these farms had previously reported issues of anthelmintic treatment failure. Despite the reporting of resistance by *Cooperia* to MLs now being a relatively common occurrence [3], cases of *Ostertagia* resistance to MLs as reported here, are much less frequent [13, 14]. Although *Cooperia* infections can potentially affect animal performance [15, 16], *Ostertagia* is still a much more significant parasite of FGS cattle, and any decline in the treatment efficacy of an anthelmintic in treating this parasitic infection can lead to significant penalties with regard to animal health, welfare and performance. It is difficult to determine what level of importance should be ascribed to the presence of *Bunostomum* spp. in the post-treatment culture of Farm 2 given that only one L_3_ larvae was detected_,_ while no L_3_ larvae of this genus were observed in the pre-treatment faecal culture. Furthermore, it is important to state that caution always needs to be exercised with regard to the interpretation of faecal larval culture results given that they may not accurately reflect the composition of the worm burden of the host animal [17]. This may be as a result of both the differences in the fecundity of the worm genera and the rates of larval mortality occurring during culture [18].

It should be recognised that a number of factors such as sample size, the detection limit of the method used to determine FEC, the pre-treatment FEC values, the level of FEC aggregation within the treated group and the method used to generate confidence intervals can influence both the detection and interpretation of treatment efficacy/inefficacy [19, 20]. In an effort to mitigate against the influence of some of these factors on both test sensitivity and specificity, 15 calves were randomly selecting for sampling on day 0. This is based on previous guidelines for conducting a FECRT (9) which states that there should be 15 animals per treatent group with a minimum individual FEC of 100 EPG. However, it was not possible to include all 15 calves in the FECR calculations on day 14 owing to insufficient rectal faecal sample sizes recovered in a few cases. The decision to conduct a FECRT using calves with low FEC values was largely based on the reluctance of the farmers in the study to leave their calves untreated for a prolonged period of time, given the potential risk of dictyocaulosis occurring under such grazing conditions [21]. It was therefore decided to use the Mini-FLOTAC technique for FEC determination as this method has a considerably lower detection limit compared to the standard McMaster method (limit of detection of 50 EPG) and this would help to offset the negative influence of the low pre-treatment FEC values in determining the true efficacy of ivermectin in this study.

A number of the parasite control practices and grazing management strategies employed by the farmers in this study favoured the development of AR. The ‘dose and move’ system [22] which was previously advocated as a parasite avoidance strategy may actually accelerate the development of AR as the only surviving nematodes that will seed the new pasture with eggs will be resistant types [23]. In addition, the use of anthelmintics on a preventive basis only and the failure to use common markers of parasitic infection such as FEC determination or measuring calf performance may result in potential overuse of anthelmintics. Indeed, an overuse of anthelmintics may hasten the development of AR by reducing the population of nematodes *in refugia*. The *in refugia* population refers to that portion of the nematode population not exposed to anthelmintic treatment [24].

The ultimate challenge in controlling nematode challenge in calves is to strike a balance between calf performance and maintaining the size of the population *in refugia*. This involves the regular monitoring of livestock throughout the grazing season for evidence of parasitism with commonly used markers such as FEC. Although FEC in general are not a reliable guide of the parasite burden of a calf as faecal egg output conforms to a stereotypic excretion pattern independent of the nature of the infection [25], whereby an initial increase in egg output is followed by a subsequent decrease which occurs logarithmically [26]. This is as a result of the fecundity of female nematodes being governed by a density-dependent mechanism which appears to involve the host animal [27]. However, it has subsequently been determined that the control of egg output in female nematodes by density-dependent mechanisms in the early stages of the grazing season appears to be minimal [28] and FEC do accurately reflect the level of challenge experienced by calves in the first two months of the grazing season. As a result, FEC measured two months post turnout are a useful tool in predicting the level of parasitic challenge in the latter half of the grazing season [28] and may potentially be used as a guide as to whether clinical parasitism may arise later in the season [29]. This can be used as a basis for determining the need for anthelmintic treatment.

## Conclusions

The detection of the presence of ML-resistant nematodes on all four farms, and in particular *Ostertagia* resistance to ivermectin on one farm, should serve as a timely reminder that greater efforts need to be made to delay the development of further resistance to commonly used anthelmintics on Irish farms. With this in mind, a more targeted approach to the control of GIN infections is advocated, providing producers are aware of the risk of dictyocaulosis occurring under these grazing conditions.

## Additional file

Additional file 1: Questionnaire used to generate the survey data. (DOCX 16 kb)
