# Supplementary material for: Ivermectin treatment failure on four Irish dairy farms
Source: Ir Vet J. 2019 May 15;72:4. doi: 10.1186/s13620-019-0142-8 (PMC6521450; doi:10.1186/s13620-019-0142-8)
Supplement: Supplementary file 1 — Questionnaire used to generate the survey data. (DOCX 16 kb) [file 13620_2019_142_MOESM1_ESM.docx]

**Questionnaire**

Farmer name:

Herd no:

Address:

Phone number:

Private Veterinary Practitioner:

**Farm Details**

1. Enterprise type and number of animal

| Dairy |  | Suckler |  | Dry Stock |  | Sheep |  | Other |  |
| --- | --- | --- | --- | --- | --- | --- | --- | --- | --- |

(2) Farm size (hectares)

________

(3) Number of first grazing season calves:

Male ___ Female________

**Calf Management**

(4) Is there a dedicated calf grazing area? Yes/No (please circle)

If no give details:

_____________________________________________________________________

(5) What size (hectares) is the calf grazing area?

_____________________________________________________________________

(6) What month are calves turned out to grass?­­­­­­­­­­­­_____________________________________________________________________

(7) Are calves turned out to pasture in batches (separate or one group)?

_____________________________________________________________________

(8) What age and weight are calves when they are turned out to grass?

___________________________________________________________________

(9) Are calves grazed as one group over the grazing season? Yes/No (please circle)

If no, please provide details: _____________________________________________

(10) Are they rotationally grazed over a number of paddocks? ___________________________________________________________________________

(11) If they are rotationally grazed, how many paddocks are in the rotation? ____________

(12) Do calves normally graze the same area (either same field or fields) each year? ____________________________________________________________________________________________________________________________________________________________________

If the answer to question 12 is no, provide details of what piece of land was used for in the previous year.

_________________________________________________________________________________________________________________________________________________________________________________________________________________________________

(13) Do calves receive concentrate feeding at grass in the first grazing season? Yes/No (please circle)

(14) If the answer to question 13 is yes, please provide details? How much concentrate per calf and when? How is concentrate given (ground/trough etc). How many troughs per calf/are they moved?

_________________________________________________________________________________________________________________________________________________________________________________________________________________________________

**Dosing of calves with wormers**

(15) Do you practice the ‘dose and move’ system (dosing calves mid season and then moving straight away into aftergrass)? ____________________________________

(16) How often are calves wormed in their first grazing season? (Time relative to turnout).

| Never |  |
| --- | --- |
| Once |  |
| Twice |  |
| Three times |  |
| Four times |  |
| Five times or more |  |

(17) If calves were wormed, did this include all or some of the calves? _______________

(18) What products were used in the previous year? _________________________________________________________________________________________________________________________________________________________________________________________________________________________________

(19) Were these treatments used as preventive treatments, curative treatments or both? ______________________________________________________________________________________________________________________________________________________

(20) When are they given? Please provide details on product and month of treatment (relative to turnout). _________________________________________________________________________________________________________________________________________________________________________________________________________________________________

(21) What product is used as the first dose? _______________________________________

(22) When is the last dose given in relation to housing? ______________________________________________________________________________________________________________________________________________________

(23) Do you rotate wormers over the course of the season? Yes/No (please circle). If yes, please give both product and details.

______________________________________________________________________________________________________________________________________________________

(24) Do you rotate wormers annually? Yes/No (please circle)

If yes, please give both products and details.

______________________________________________________________________________________________________________________________________________________

(25) If you do not rotate wormers annually, when was the last time you changed product? Please give both product and details.

______________________________________________________________________________________________________________________________________________________

(26) In terms of parasite monitoring during the grazing season, are growth rates measured and used as a guide for parasite burden? _________________________________________

(27) Do you routinely collect dung samples (≥ once a year) for egg counting in order to measure parasite burden? _____________________________________________________

(28) Is there a separate worming policy for spring and autumn born calves if applicable? Yes/No (please circle)

If yes please specify and give details.

__________________________________________________________________________________________________________________
